# The time to decline: tracing a cohort’s descendants in below replacement populations

**DOI:** 10.1186/s41118-018-0026-x

**Published:** 2018-01-23

**Authors:** Robert Schoen

**Affiliations:** Pennsylvania State University, San Francisco, CA USA

**Keywords:** Replacement level, Below replacement, Generational succession, Population projection, Population decrease

## Abstract

A number of contemporary populations are exhibiting sustained fertility at levels substantially below long-term replacement. Nonetheless, relatively few populations are actually diminishing in size. Here, we approach that apparent paradox by analyzing the time before the number in a birth cohort, and its descendants, falls below the initial number in the cohort. First, models are examined with constant below replacement fertility, cohort extinction at age 75 or 85, and no mortality below the highest age attained. For a net reproduction rate (NRR) of 0.75, it takes 150 years for the cohort’s descendants to be fewer than the cohort’s original size if persons live to age 85, and over 130 years if persons live to age 75. If the NRR is at least 0.60, it takes a century before the descendants are fewer in number than the original cohort. Second, projections are done for the USA 2012, Italy 2012, and Hong Kong 2011 assuming that fertility and mortality remain constant. The results resemble the projections. For example, in Italy, with actual mortality and an NRR of 0.70, it takes over 125 years before the descendants of a cohort are fewer in number than the initial cohort. A relatively simple equation for the long term “time to decline” is presented, showing that it depends primarily on the level of fertility, secondarily on longevity, and only modestly on the mean age of fertility.

## Introduction

Replacement level is when, on average, every woman has one daughter. The net reproduction rate (NRR), the average number of daughters a cohort of women bears subject to a given set of fertility and mortality rates, is then 1. Over the last three decades, below replacement fertility has spread to characterize most of Europe, overseas Europe, and East Asia. For the 2010–2015 period, the United Nations Population Division estimated that Europe had an NRR of 0.763, below replacement since 1975–1980; Eastern Asia an NRR of 0.699, below replacement since 1990–1995; and Northern America an NRR of 0.896, below replacement since 1970–1975. The Chinese Autonomous Region of Macau has the lowest fertility of any listed entity, with an NRR of 0.577 (UN Population Division 2015, File FERT/5). In contrast, only Germany, Japan, and some countries in Southern and Eastern Europe have a negative rate of natural increase (i.e., more deaths than births). The largest rate of natural decrease, in Bulgaria 2010–2015, was only 0.57% per year, a rather slow rate of decline (UN Population Division 2015, File POP/3).

The explanation for the broad prevalence of below replacement fertility and the modest extent of negative natural increase is population momentum, an important concept introduced in Keyfitz (1971). Population momentum is the factor an initial population will grow (or decline) after it has an immediate shift to replacement level fertility. In general, growing populations tend to continue growing because they have large cohorts at the reproductive ages and small cohorts at older ages. Preston (1986) examined growing populations that experienced a fall in fertility to replacement level and found that, over the transition to zero growth, the population under age 30 remained virtually constant, the population between the ages of 30 and 60 increased by a factor approximately equal to the pre-shift NRR, and the population 60 to 90 increased by a factor approximately equal to the square of that NRR. An in-depth discussion of population momentum can be found in Schoen (2006, Chap. 3).

Momentum, however, is a population level concept that does not provide a cohort level perspective or explicitly consider the descendants of a cohort. Here, we do so, starting with a birth cohort and tracing its descendants over time. The goal is to determine, in terms of a fixed level and pattern of below replacement fertility, how many years pass before the sum of the surviving members of the cohort, and the number of its living descendants, falls below the initial number in the cohort.

## Projecting a cohort and its descendants in a simplified model

The first approach used here projects a birth cohort and its descendants in the usual interval-by-interval manner. We then derive an analytical procedure for projecting the model population to any future point. The principal goal is to find the time at which total population size falls below the number in the initial cohort.

### The simplified projection model

Let the initial population consist only of females in the first age group, and consider only female births. Assume that age-specific fertility remains constant over time and that the population is closed to migration. For simplicity, to focus on fertility, and to recognize the low mortality prevailing in most contemporary below replacement populations, assume that there is no mortality below the highest age attained, with the cohort becoming extinct on attaining that age. In separate calculations, we take that highest age to be 75 or 85 years.

The standard cohort-component approach to population projection advances an initial population using a Leslie matrix, i.e., a projection matrix that has fertility values in its first row and survival probabilities on its subdiagonal (Preston et al. 2001, Chap. 6). While projections have become quite sophisticated (cf. Sevcikova et al. 2016), here, we use a basic approach that proceeds using 5-year age and time intervals and continues past the point where the total number in the population is less than the initial number in the cohort.

### The model population projection procedure

Projections were carried out for NRR levels from 0.50 to 1.0 and for fertility patterns with a mean age of 25, 29, and 33 years. Those bounds span the range of fertility levels and patterns in most below replacement fertility populations.

A base fertility pattern, roughly following patterns in the USA during the late 20th century, was created for each mean age of fertility, and those patterns are shown in Appendix Table 4. In each base pattern, the sum of the fertility values is one.

To facilitate the analytical model, the base age-specific fertility rates were adjusted to the desired NRR by a Sykes transformation (Sykes 1973), an approach that was also employed to adjust fertility values in the Coale and Trussell (1974) model fertility schedules. If the base fertility value for the *j*th age group is *f*_*j*_, then the Sykes transformed fertility value, *F*_*j*_, is

1 $$ {F}_j={f}_j\;\exp \left(5 rj\right) $$

where the time interval is 5 years and *r* is the intrinsic annual rate of natural increase associated with the *F*_*j*_. Index *j* reflects the sequence of the 5-year age groups, with *j* = 1 for ages 0–4, *j* = 2 for ages 5–9, and so on. The NRR and *r* are related by Lotka’s equation (Schoen 2006, p. 11)

2 $$ \mathrm{NRR}=\exp \left(5 rT\right) $$

where *T* is Lotka’s mean length of generation in units of 5 years. The value of *T* is close to the mean age of fertility, *μ*, also in units of 5 years, which is given by

3 $$ \mu ={\Sigma}_jj\;{f}_j $$

where the sum over age categories *j* spans all ages of fertility. Equation (3) indicates that the value associated with an age group is the age at the end of the age interval.

Keyfitz (1977, p. 126) expressed *T* in terms of a series in the moments of the *f*_*j*_. In low fertility populations, the mean and variance of fertility are often roughly equal, and that approximate relationship is appropriate here as it simplifies the equations while having little effect on the results. Up to second moments, the Keyfitz series can then be written

4 $$ T=\mu \left(1\hbox{--} r/2\right) $$

Using Eqs. (2)–(4), the modified version of Lotka’s solution for *r* in terms of the NRR and *μ* (in 5-year units) can be written

5 $$ r=1\hbox{--} {\left[1\hbox{--} 2\;\ln\;\mathrm{NRR}/\left(5\mu \right)\right]}^{\raisebox{1ex}{$1$}\!\left/ \!\raisebox{-1ex}{$2$}\right.} $$

where ln designates the natural logarithm (Schoen 2006). Equations (1)–(5) allow the Sykes transformation to be implemented for all *μ* and NRR values.

### An analytical alternative to interval-by-interval projection

In the model described above, long-term projections can be made analytically, as well as interval by interval. To do so, we now derive an expression for total population size at any time point based on two parameters, *μ* and *r*.

Let the constant population projection (Leslie) matrix that takes the population from any time *t* − 1 to time *t* be denoted by **A**. Here, with a life expectancy at birth [*e*(0)] of 85 years, **A** is a 17 × 17 matrix that reflects the age groups (0,5) through (80,5). Then, under the Sykes transformation, **A** can be expressed as

6 $$ \mathbf{A}=\exp (5r)\;{\mathbf{U}}_{\mathbf{d}}\;\mathbf{F}\;{\mathbf{V}}_{\mathbf{d}} $$

where **F** is a row stochastic Leslie-form matrix with ones on the subdiagonal and the *f*_*j*_ of Eqs. (1) and (3) as the elements of the first row (Schoen 2006, Chap. 7). At ages below 15 and over 50, the *f*_*j*_ are zero. The matrix **U**_**d**_ is a 17 × 17 diagonal matrix whose *j*th diagonal element is exp(− 5*r*[*j* − 1]), and **V**_**d**_ is the inverse of **U**_**d**_.

The 17 × 17 product matrix **P(0,t)** that takes the initial (time 0) population to time *t* can then be written

7 $$ \mathbf{P}\left(\mathbf{0},\mathbf{t}\right)={\mathbf{A}}^{\mathbf{t}}=\exp \left(5 rt\right)\;{\mathbf{U}}_{\mathbf{d}}\;{\mathbf{F}}^{\mathbf{t}}\;{\mathbf{V}}_{\mathbf{d}} $$

For a sufficiently large *t*, an interval whose length is examined below, matrix **F**^**t**^ becomes a rank one matrix, i.e., it can be represented as the product of a column vector, **u**, and a row vector, **v'** (Schoen 2006, p. 28). Here, *e*(0) = 85, there is no mortality during the first 17 age intervals, and **F** is consistent with zero growth. Hence 17 × 1 column vector **u** is a vector of ones. The 1 × 17 row vector **v'** has first element 1/*μ* and *j*th element Σ_*i* = *j*_ *f*_*i*_/*μ* (Schoen 2006, p. 157, #2b).

If the initial population has one female in the first age group, the initial population vector, **x**_**0**_, is a 17 × 1 column vector with first element 1 and all other elements zero. The population at sufficiently large time *t* ≥ 17, **x**_**t**_, is then given by

8 $$ {\mathbf{x}}_{\mathbf{t}}=\mathbf{P}\left(\mathbf{0},\mathbf{t}\right)\;{\mathbf{x}}_{\mathbf{0}}=\exp \left(5 rt\right)\;{\mathbf{U}}_{\mathbf{d}}\;{\mathbf{F}}^{\mathbf{t}}\;{\mathbf{V}}_{\mathbf{d}}\;{\mathbf{x}}_{\mathbf{0}}=\exp \left(5 rt\right)\;{\mathbf{U}}_{\mathbf{d}}\;\mathbf{u}\;{\mathbf{v}}^{\hbox{'}}\;{\mathbf{V}}_{\mathbf{d}}\;{\mathbf{x}}_{\mathbf{0}} $$

Using the relationships noted above, (**v' V**_**d**_ **x**_**0**_) is the scalar (1/*μ*), and Eq. (8) simplifies to

9 $$ {\mathbf{x}}_{\mathbf{t}}=\exp \left(5 rt\right)\;\mathbf{w}/\upmu $$

where 17 × 1 column vector **w** = (**U**_**d**_ **u**) has first element 1 and *j*th element exp(− 5*r*[*j* − 1]).

Using Eq. (9), the total population at time *t*, *P*_T_(*t*), can be expressed as

10 $$ {P}_{\mathrm{T}}(t)={\mathbf{1}}^{\hbox{'}}\;{\mathbf{x}}_{\mathbf{t}}={\mathbf{1}}^{\hbox{'}}\;\mathbf{w}\;\exp \left(5 rt\right)/\mu =\mathrm{c}\;\exp \left(5 rt\right)/\mu $$

where **1'** is a 1 × 17 row vector of ones and c = **1' w**. Scalar c is the sum of a 17-term geometric series with initial value one and fixed ratio exp(− 5*r*). The constant sum of that series can be written (*r* ≠ 0) as

11 $$ \mathrm{c}={\mathbf{1}}^{\hbox{'}}\;\mathbf{w}=\left(1\hbox{--} \exp \left(\hbox{--} 85r\right)\right)/\left(1\hbox{--} \exp \left(\hbox{--} 5r\right)\right) $$

Combining Eqs. (10) and (11), the total population at time *t* is given by

12 $$ {P}_{\mathrm{T}}(t)=\left[\left(1\hbox{--} \exp \left(\hbox{--} 85r\right)\right)/\left(1\hbox{--} \exp \left(\hbox{--} 5r\right)\right)\right]\ \left[\exp \left(5 rt\right)/\mu \right] $$

Thus, in the long term, Eq. (12) shows that total population size at time *t* is a constant factor, c/*μ*, times a decreasing exponential term in negative *r*. The constant factor represents the size of the implicit initial stable population divided by the mean age of fertility (approximately the length of a generation).

Of particular interest here is the time t*, when *P*_T_(t*) = 1. If the initial cohort lives 85 years, t* must be at least 85 years. To find an expression for t*, note that Eqs. (10) and (11) yield

13 $$ {P}_{\mathrm{T}}\left({t}^{\ast}\right)=1=\mathrm{c}\;\exp \left(5{rt}^{\ast}\right)/\mu $$

which gives, with t* and *μ* in units of 5 years,

14 $$ {t}^{\ast }=\ln \left(\mu /\mathrm{c}\right)/(5r) $$

Thus, in the long term, time to decline t* can be expressed in terms of two parameters, the mean age of fertility, *μ*, and the intrinsic growth rate, *r*, with Eq. (5) relating *r* to *μ* and the NRR.

## The results for the simplified projection model

### The interval-by-interval projection results

Table 1 shows the results for the time to decline (t*) projections for NRRs of 0.50 to 1, separately for mean ages of fertility of 25, 29, and 33 years and for life expectancies of 75 and 85 years. For times greater than *e*(0) years, linear interpolation was used to determine t* within the 5-year projection intervals.

**Table 1:** Time to decline (t*) values, in single years, by level and mean age of fertility

NRR	*μ* = 25 years			*μ* = 29 years			*μ* = 33 years		
	*e*(0) = 75 years	*e*(0) = 85 years		*e*(0) = 75 years	*e*(0) = 85 years		*e*(0) = 75 years	*e*(0) = 85 years	
	Projection	Projection	Eq. (14)	Projection	Projection	Eq. (14)	Projection	Projection	Eq. (14)
1.00	∞	∞	∞	∞	∞	∞	∞	∞	∞
0.95	571.5	637.7	637.7	573.1	649.1	649.0	564.0	649.6	649.6
0.90	297.2	332.3	332.2	297.9	337.6	337.6	293.3	337.8	337.8
0.85	206.4	230.5	230.1	206.3	234.1	234.1	203.9	233.9	234.1
0.80	161.8	179.7	180.4	160.8	182.6	182.6	158.5	182.9	182.4
0.75	135.5	151.4	150.4	133.4	152.0	151.9	134.6	151.3	151.6
0.70	117.7	130.2	130.6	116.4	132.1	131.6	110.2	127.1	131.2
0.65	99.2	112.5	116.7	101.4	114.7	117.3	103.9	116.7	116.8
0.60	95.6	106.6	106.4	96.4	108.0	106.7	97.8	110.8	106.1
0.55	91.8	102.7	98.6	75	102.7	98.7	75	85	98.0
0.50	75	85	92.6	75	85	92.4	75	85	91.6

Note: The time to decline is the time point when the total number in the population is less than the size of the initial cohort. Mean age of fertility *μ* is calculated from Eq. (3)

There are huge differences in the time to decline by NRR level. The higher the NRR, the larger t*. For NRRs of 0.65, t* is a century or more; for NRRs of 0.75, t* is 133 or more years; and for NRRs of 0.95, t* is well over five centuries. Differences by mean age of fertility are present, but relatively small. There are no “tempo” effects, as fertility is held constant.

As to be expected, the time to decline is less when life expectancy is 75 than when it is 85 years. Proportionally, however, the effect of a 10-year smaller *e*(0) is not all that large relative to t*. When the NRR is 0.95, the difference in t* is some 65 to 85 years (10–13%), while when the NRR is 0.75, differences are about 15 to 20 years (again 10–12%).

Equation (14) suggests that a higher mean age of fertility is associated with a longer time to decline. However, parameter c is a function of *r*, which in turn is a function of *μ*; hence, a change in *μ* can introduce offsetting changes in *r* and c. Table 1 shows that while there is a tendency for t* to increase when the mean age of fertility rises, there are many exceptions to that pattern.

With a life expectancy of 85 years, when the NRRs are 0.50 (or 0.55 when *μ* = 33), t* is at its minimum, i.e., 85 years. By iterating on Eq. (13), we can find NRR*, the fertility level at which the total population at time 85 years is one. The results indicate:

**Table:** 

*μ* (in years)	NRR*	TFR*
25	0.534	1.09
29	0.555	1.14
33	0.591	1.21

where TFR* is the corresponding total fertility rate (TFR). The TFR is set equal to 2.05 times the NRR, using the customary sex ratio at birth of 105 males per 100 females. A TFR of 1.3, that is women having an average of 1.3 children, is considered very low fertility, but even sustained fertility at that level implies a time to decline of about 110 years.

### The analytical results

Analytical projections using Eq. (14) are also presented in Table 1 for *e*(0) = 85. The equation-based values implicitly assume that the population is approximately stable, which initially is far from the case. Stability takes several generations to arise, even more when one begins with a single cohort, and is slower for higher mean ages of fertility.

For NRRs of 0.70 or above (0.75 when *μ* = 33 years), the equation-based t* values are quite close to the projections. For NRRs less than 0.60, the Eq. (14) values are poor.

### The trajectory of a cohort

For observed populations, a rough rule of thumb is that the population becomes approximately stable up to age *x* after about (60 + *x*) years (Schoen 2006). Here, we begin with a single cohort age 0, and Table 1 suggests that total population size is not roughly stable for up to 150 years. At the 150-year time point, growth is approximately stable (i.e., equal to exp(5*r*)), though projected and stable values still differ by 1½–3% when *μ* = 33 years.

**Fig. 1:**
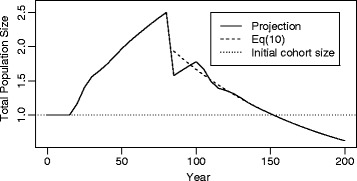
The total size of a cohort and its descendants, by projection and by Eq. (10), for NRR = 0.75 and mean age of fertility of 29 years

To get a fuller sense of the trajectory of the total size of a cohort and its descendants, one can project total population size over time and compare it to the analytical trajectory provided by Eq. (10). For *e*(0) = 85, Fig. 1 and Table 2 show such a comparison for NRR = 0.75 and *μ* = 29 years, values that are in the middle of the ranges considered here. The equation-based trajectory begins at time 85 years, as that is the first post initial cohort time point.

**Table 2:** The total size of a cohort and its descendants, as calculated by projection and by Eq. (10), for NRR = 0.75 and *μ* = 29 years

(1)	(2)	(3)	(4)	(5)
Time (*t*) [years]	Projected total size [*P*_T_(*t*)]	Growth rate [*P*_T_(*t*)/*P*_T_(*t* − 5)]	Total size from Eq. (10)	Proportional error
0	1.0			
5	1.0	1.0		
10	1.0	1.0		
15	1.0	1.0		
20	1.1642	1.1642		
25	1.4064	1.2081		
30	1.5625	1.1110		
35	1.6475	1.0544		
40	1.7418	1.0572		
45	1.8534	1.0641		
50	1.9695	1.0626		
55	2. 0730	1.0526		
60	2.1651	1.0444		
65	2.2544	1.0412		
70	2.3417	1.0387		
75	2.4244	1.0353		
80	2.5023	1.0321		
85	1.5763	0.6300	1.9352	− 0.2277
90	1.6474	1.0451	1.8420	− 0.1181
95	1.7153	1.0412	1.7533	− 0.0221
100	1.7796	1.0375	1.6689	0.0622
105	1.6764	0.9420	1.5885	0.0524
110	1.4923	0.8902	1.5120	− 0.0132
115	1.3916	0.9325	1.4392	− 0.0342
120	1.3594	0.9769	1.3699	− 0.0077
125	1.3153	0.9675	1.3039	0.0087
130	1.2515	0.9515	1.2411	0.0083
135	1.1809	0.9436	1.1813	− 0.0004
140	1.1206	0.9490	1.1244	− 0.0034
145	1.0698	0.9546	1.0703	− 0.0005
150	1.0197	0.9532	1.0187	0.0009
155	0.9697	0.9510	0.9697	0.000 03
160	0.9225	0.9513	0.9230	− 0.000 54
165	0.8785	0.9523	0.8785	− 0.000 06
170	0.8366	0.9524	0.8362	0.000 48
175	0.7962	0.9516	0.7960	0.000 26
180	0.7574	0.9514	0.7576	− 0.000 23
185	0.7209	0.9518	0.7211	− 0.000 28
190	0.6864	0.9521	0.6864	0.000003
195	0.6535	0.9520	0.6534	0.000 17
200	0.6219	0.9518	0.6219	0.000 08

Notes: The initial cohort is age 0 at time 0. The 5-year stable growth rate is 0.9518. The proportional error in the Eq. (10) analytical projection in column (5) is calculated as [(2) − (4)]/(2), referring to columns (2) and (4)

Column (2) of Table 2 shows that for the first three time periods, before the cohort reaches age 15, the projected total population remains at 1. Between times 15 and 80 years, population size steadily increases, as the cohort and its offspring reproduce. At time 85 years, when the initial cohort has just died, total population size drops by nearly 1. After that point, Fig. 1 shows how total population size fluctuates in waves of diminishing amplitude around the stable population size given by the Eq. (10)-based decreasing exponential [the plotted values are shown in column (4) of Table 2].

Column (3) presents the interval by interval population growth rates. After some 130 years, the growth rate of the projected population becomes quite close to the ultimate stable population growth rate of 0.9518. Column (5) compares the Leslie and analytical projections, showing the proportional error in the Eq. (10) projection. The direction of the error varies because of the fluctuations in the growth rate of the projected population. After 120 years, the two trajectories are within 1% of each other, with the difference inconsequential after 145 years.

## Projecting three observed populations

It is worth exploring the time to decline using the vital rates observed in contemporary below replacement populations. Table 3 shows the results of such projections for the USA 2012, Italy 2012 (with the death rates of 2015), and Hong Kong 2011, geographically dispersed populations with a range of low fertility rates and high life expectancies.

**Table 3:** The time to decline based on observed rates in three contemporary populations

Measure	USA 2012	Italy 2012*	Hong Kong 2011
Life expectancy at birth	81.2	84.6	86.7
Total fertility rate	1.88	1.43	1.56
Net reproduction rate	0.92	0.70	0.76
Mean age of fertility	28.6	31.4	31.2
Variance of age of fertility	38.3	33.4	30.7
Time to decline (t*)	341.7	126.7	157.5
Ratio of total population size to initial cohort size at year:			
100	2.28	1.35	1.64
150	1.93	0.79	1.08
200	1.62	0.43	0.68
250	1.36	0.24	0.44
300	1.14	0.14	0.28

Sources: Fertility data from United Nations *Demographic Yearbook 2013*, Table 10 (United Nations Statistical Division 2014). Female mortality values for the United States from Arias et al. (2016), Table 3; for Italy from ISTAT (2016), Life Tables of the Resident Population—2015; for Hong Kong from Hong Kong Life Table for Females, 2011, Table 3, Hong Kong Central Statistics Dept

Notes: *Italian mortality is based on the 2015 Italian Life Table. In all cases, the sex ratio at birth is taken to be 105 males per 100 females. See discussion in text

The USA 2012, with an NRR of 0.92, requires over 340 years before the descendants of a cohort are fewer than the original number in the cohort. Even at time 200, the descendants of the initial cohort are 62% more numerous. Hong Kong 2011, with an NRR of 0.76, has a time to decline over 155 years, but at time 200, the descendants of the initial cohort number only about two thirds the size of the initial cohort. Italy 2012, with an NRR of 0.70, has a time to decline of just over 125 years, and at time 200, the descendants of the original cohort number only 43% of their initial number.

The introduction of actual mortality rates seems to have only a small effect, as the observed population rates yield times to decline quite similar to those in Table 1. For example, Italy has a time to decline of 126.7 years with an NRR of 0.70, a life expectancy of 84.6 years, and a mean age of fertility of 31.4 years. For an NRR of 0.70 and a life expectancy of 85 years, Table 1 has a time to decline of 132.1 years for *μ* = 29 years and 127.1 years for *μ* = 33 years.

## Summary and conclusions

Population momentum means that a growing population continues to increase in size for some years after its fertility falls to replacement. Here, we take a birth cohort, individual level, perspective, and examine how long it takes, under below replacement fertility, before the number of survivors of a cohort, plus its living descendants, falls below the number in the initial cohort. With no mortality below the highest age attained (75 or 85), and constant below replacement fertility, we find that t*, the time to decline, varies dramatically with the level of fertility, moderately with longevity, and modestly with the mean age of fertility.

As the cohort reproduces, the succession of generations prolongs the onset of population decrease. When *e*(0) is 85 and the NRR is 0.95, it takes nearly 650 years for total population size to be less than 1. For an NRR of 0.50 or below, t* is 85 years, the point at which the initial cohort dies. Projections with the observed vital rates of three contemporary below replacement populations suggest that the model simplifications have only a minor effect on the time to decline.

Analytically, Eq. (10) allows a simplified projection in terms of life expectancy, the NRR, and the mean age of fertility. That procedure tracks the projected population size quite closely after about 150 years.

Even with an NRR of 0.65, an *e*(0) of 75, and no migration, the time to decline is almost a century. A cohort produces its own momentum, propelled by the arrival of succeeding generations.

## Appendix

**Table 4:** Age-specific fertility values (*f*_*j*_) for three mean ages of fertility (*μ*)

Age	Mean age of fertility (*μ*)		
	25	29	33
15–19	0.360	0.200	0.060
20–24	0.430	0.310	0.180
25–29	0.110	0.210	0.250
30–34	0.070	0.120	0.240
35–39	0.015	0.100	0.160
40–44	0.010	0.050	0.100
45–49	0.005	0.010	0.010
All ages	1.000	1.000	1.000

Note: The mean ages are calculated based on age at the end of each age interval, per Eq. (3). That mean age is also the mean implied by the dominant left eigenvector of matrix **F** in Eq. (6)
